# Application of CAD-CAM in Dentistry

**DOI:** 10.6026/973206300200547

**Published:** 2024-05-31

**Authors:** Niladri Maiti, Niva Mahapatra, Dhruvi Patel, Jeel Chanchad, Anushka Saurabhbhai Shah, SK Mahboob Rahaman, Pratik Surana

**Affiliations:** 1 School of Dentistry, Central Asian University, Tashkent, Uzbekistan; 2 Department of Oral and Maxillofacial Pathology, Kalinga Institute of Dental Sciences, KIIT Deemed to be University, Bhubaneswar, Odisha, India; 3 Dhanak Dental Hospital, Ahmedabad, Gujarat, India; 4 Rutgers School of Public Health, New Jersey, USA; 5 Health information management, George Brown College, Toronto, Canada; 6 Department of Conservative Dentistry & Endodontics, North Bengal Dental College & Hospital, Darjeeling, West Bengal - 734012, India; 7 Department of Pedodontics and Preventive Dentistry, Maitri College of Dentistry and Research Centre, Durg, Chhattisgarh, India

**Keywords:** CAD-CAM, Dentistry, application

## Abstract

The application of CAD-CAM (Computer-Aided Design and Computer-Aided Manufacturing) technology has become increasingly prevalent in dentistry in recent years. Dental restorations are designed and created using CAD-CAM by enhancing the precision and efficiency. Customization of dental prostheses such as crowns, veneers, inlays, onlays and bridges is possible with CAD-CAM.

## Background:

The terms "computer-aided design" and "computer-aided manufacturing" (CAD/CAM) describe the use of computer software for prosthesis design and fabrication. This technology has transformed dentistry and had an impact on all areas of prosthetic and restorative dentistry. [1] The integration of CAD-CAM in dentistry began in the 1980s and has since undergone significant advancements. Traditional methods of creating dental restorations typically involved manual crafting, that is time-consuming and often less accurate. [2] With the advent of CAD-CAM, dental professionals can bypass these limitations by utilizing digital impressions and automated manufacturing processes. [3]Principle of CAD-CAM: The principle of CAD-CAM in dentistry can be understood in a three-stage process. [4] The first stage involves capturing the dental anatomy through optical scanning to create a digital impression. [5] The second stage involves the use of CAD software where the practitioner designs the restoration on a computer, ensuring a precise fit and desired esthetics. [6] The last stage utilizes CAM, where the design is sent to a milling machine or a 3D printer that fabricates the restoration from a block of ceramic or other materials. [7]

## Various application of CAD-CAM: 

## Design and fabrication of dental crowns:

CAD-CAM technology streamlines the creation of dental crowns, allowing accurate measurements and precise milling, resulting in crowns that fit better and require less adjustment. [8]

## Dental bridges construction:

Similar to crowns, bridges can be designed using CAD software, ensuring a seamless fit over the adjacent teeth to replace missing ones. The CAM process then mills the bridge from appropriate materials. [9]

## Inlays and onlays:

For teeth that require a conservative restoration, inlays and onlays designed using CAD-CAM provide a durable and aesthetic solution. [1]They offer a more precise fit than traditional fillings. [2]

## Veneers:

CAD-CAM allows for detailed design and fabrication of dental veneers, which cover the front surface of teeth. [4] The result is a more natural and aesthetically pleasing appearance. [5]

## Orthodontic appliances:

Clear aligners and retainers can be designed using CAD software based on digital impressions. [3] CAM then produces these appliances using suitable biocompatible materials. [2]

## Implant planning and components:

CAD-CAM is used in designing and fabricating dental implant components, including custom abutments that ensure a proper fit and optimal esthetics. [1,6]

## Full and partial dentures:

Digital design of dentures enhances their comfort and function. CAD-CAM technology can also be used to mill or print denture bases and teeth. [10]

## Nightguards and mouthguards:

Using CAD-CAM technology to design and manufacture nightguards and mouthguards results in a product that provides protection and a comfortable fit. [10]

## Removable dental appliances:

Other removable appliances, such as snore guards or removable partial dentures, can be accurately fabricated with CAD-CAM technology, allowing for a better patient experience. [7]

## Jaw and dental models:

Accurate replicas of a patient's mouth, including the teeth and surrounding structures, can be created using CAD-CAM for diagnostic purposes or treatment planning. [8]Each application leverages the accuracy and efficiency of CAD-CAM technology to improve the dental restoration process, resulting in better outcomes and more satisfactory patient experiences. [9] Continued innovation is expected to further enhance these applications, incorporating new materials and techniques. [5]

## Advantages of CAD-CAM in dentistry Efficiency and time saving:

CAD-CAM technology enables dentists to create restorations within a few hours, which traditionally would have taken weeks. [10]

## Precision:

The digital impressions and automated manufacturing result in highly precise and well-fitting restorations. [11]

## Customization:

It allows for a high degree of customization, aiding in patient-specific solutions and better cosmetic outcomes. [4]

## Digital storage:

The 3D models created using CAD can be stored digitally, saving physical storage space and allowing easy retrieval for future reference. [6]

## Reduced human error:

Minimizes the potential for human error compared to traditional methods. [7]

## Materials:

A wide range of materials can be used in the process, such as ceramics, composite resin, and metals, allowing dental professionals to choose the appropriate material for each specific case. [8]

## Limitations of CAD-CAM in Dentistry Cost:

The initial investment for CAD-CAM equipment is very high, which can be a barrier for smaller practices. [11]

## Training:

Dentists and staff require extensive training to use these systems effectively. [12]

## Maintenance:

The machines require regular maintenance and updates to ensure they function properly. [13]

## Material limitations:

Although there are many materials available, some specific dental materials can still be challenging to sculpt using CAD-CAM systems. [14]

## Technical Issues:

Glitches and hardware failures can interrupt the workflow, necessitating immediate technical support. [15]

## Various materials used for CAD-CAM:

The materials used in CAD-CAM dentistry have evolved, offering advantages such as improved aesthetics, durability, and biocompatibility.

## Some of the common materials used in CAD-CAM dentistry:

## Adhesive ceramics:

The first materials designed specifically for CAD/CAM systems were glass-ceramics. [16] Because they contain a large proportion of glass, they are among the most transparent and beautiful materials. This gives the restoration a "chameleon" aspect that lets it match the colour of the original tooth. [17- 18]

## Hybrid ceramics:

A brand-new class of CAD/CAM chairside materials called hybrid ceramics was created to capitalise on the distinct visual qualities of ceramic materials along with the enhanced fracture resistance and decreased fragility of composite resins. [19] It has been shown that hybrid ceramics can be machined more easily and don't require extra heat cycles. Additionally, they have a good bending resistance and can be employed at thinner thicknesses. [20-21]

## Lithium disilicates:

A significant advancement in the realm of fixed prosthesis was the invention of glass ceramics with enhanced resistance qualities. [22] In comparison to earlier adhesive glass ceramics, IPS e.max CAD (IvoclarVivadent) has a fracture resistance that is noticeably higher and a bending resistance of over 350 MPa. It was first released in 2006. For partial adhesive restorations including veneer and onlays, overlays, and crown restorations, lithium disilicate is recommended. [23] Additionally, lithium disilicate has shown promise as a single implant restoration crown and as a single hybrid implant abutment supported by titanium tibase. [23-24]

## Zirconia:

Although zirconia is a heterogeneous polycrystalline ceramic, it is resistant to conventional acid biting techniques and has good mechanical qualities (flexural strength 500-1200 MPa, elastic modulus of 210 GPa).[25] It also has acceptable aesthetic qualities. For zirconia, the reported resistance to the fracture force is greater than 1000 MPa. Among the many integral ceramics, it has the lowest rate of wear against the antagonist, superior biocompatibility, and decreased plaque retention as compared to titanium, both in vivo and in vitro. [26]Each material has specific indications based on the patient's clinical situation, aesthetic needs, and the restoration's location in the mouth. The choice of material can affect the longevity, appearance, and functionality of the dental restoration. CAD-CAM technology has significantly expanded the capabilities of dental professionals to provide restorations that are not only highly aesthetic but also precisely tailored to each patient's unique dental anatomy. [27]

## Future directions:

The future of CAD-CAM in dentistry appears promising, with continual advancements in technology that could broaden its applications and improve its accessibility. [12,13] The implementation of artificial intelligence and machine learning in CAD-CAM systems could further enhance design efficiency and predictive outcomes, propelling dentistry into a new era of digital innovation.[14,15]

## Conclusion:

CAD-CAM technology has revolutionized the dental industry by enhancing the ability to design and manufacture dental restorations with exceptional precision and efficiency. While embracing this innovative technology, professionals have enjoyed the flexibility to deliver dental care with increased customization and improved aesthetic outcomes. However, the high cost of acquisition and operation, steep learning curve, and potential technical challenges are barriers that must be weighed carefully. Despite these limitations, the advantages of CAD-CAM technology are driving its increasing adoption, signaling a future where digital dentistry could become the standard for delivering patient care. As technological advancements continue, it is likely that CAD-CAM will become more accessible, user-friendly, and integrated within dental practices, further enhancing its application and benefits in dental care.
